# Major depression and household food insecurity among individuals with multidrug-resistant tuberculosis (MDR-TB) in South Africa

**DOI:** 10.1007/s00127-019-01669-y

**Published:** 2019-02-13

**Authors:** Andrew Tomita, Suvira Ramlall, Thirusha Naidu, Sbusisiwe Sandra Mthembu, Nesri Padayatchi, Jonathan K. Burns

**Affiliations:** 1 KwaZulu-Natal Research Innovation and Sequencing Platform (KRISP), College of Health Sciences, University of KwaZulu-Natal, Durban, South Africa; 2 Centre for Rural Health, School of Nursing and Public Health, University of KwaZulu-Natal, Private Bag X7, Congella Durban, 4013 South Africa; 3 Department of Psychiatry, University of KwaZulu-Natal, Durban, South Africa; 4 Department of Behavioural Medicine, University of KwaZulu-Natal, Durban, South Africa; 5 Wentworth Hospital, KwaZulu-Natal Department of Health, Durban, South Africa; 6 MRC HIV-TB Pathogenesis and Treatment Research Unit, Centre for the AIDS Programme of Research in South Africa (CAPRISA), Durban, South Africa; 7 Institute of Health Research, University of Exeter, Exeter, UK

**Keywords:** Food insecurity, Drug-resistant tuberculosis, Depression, South Africa

## Abstract

**Purpose:**

Household food insecurity in South Africa is a pervasive public health challenge. Although its link to chronic health conditions is well established, its relationship to mental illness, particularly major depression, is not well-understood. Despite KwaZulu-Natal Province being the epicenter of the drug-resistant tuberculosis (MDR-TB) epidemic, and having the largest share of poverty in South Africa, this relationship remains unexamined. This study investigated the association between major depressive episode (MDE) and household food insecurity among individuals with MDR-TB.

**Methods:**

We enrolled and interviewed 141 newly admitted microbiologically confirmed MDR-TB inpatients at a specialized TB hospital in KwaZulu-Natal Province, South Africa. Logistic regression models were fitted to assess the relationship between MDE and household food insecurity, while accounting for socio-demographic status (e.g., age, gender, education, marital status, social grant status, income, and preference for living in one’s community).

**Results:**

The prevalence of MDE and household food insecurity was 11.35% and 21.01%, respectively. MDE was significantly associated with household food insecurity (aOR 4.63, 95% CI 1.17–18.38). Individuals who are female (aOR 6.29, 95% CI 1.13–35.03), young (aOR 8.86, 95% CI 1.69–46.34), have low educational attainment (aOR 6.19, 95% CI 1.70–22.59) and receive social grants (aOR 7.60, 95% CI 2.36–24.48) were most at risk of household food insecurity.

**Conclusions:**

MDE in individuals with MDR-TB was significantly associated with household food insecurity, independent of socio-economic status. Although MDR-TB is not exclusively a disease of the poor, individuals from socio-economically disadvantaged backgrounds (e.g., female, young adults, low education, and social grant recipients) were more likely to experience household food insecurity. Our study underscores the need to address the co-occurring cycles of food insecurity and untreated MDE in South Africa.

## Introduction

Despite the political advances in South Africa since the transition to democracy, there has been little progress in addressing household food insecurity (21.3% in 2017 versus 21.2% in 2011 [1]). Food security, defined as access by all people at all times to enough food for an active and healthy life [2], is a cornerstone of development [3], and an indispensable part of well-being [4], including mental well-being [5, 6]. Food insecurity is a quintessential symbol of poverty, with an increasing proportion of South Africans living under the food poverty line (25.2% in 2015 versus 21.4% in 2011 [7]). KwaZulu-Natal (KZN) Province has the second largest population in South Africa (11.4 million people in 2018 [8]), with a high unemployment rate of 40.9% (in 2018) [9], and a secondary education level of 68.2% (in 2016) [10]. Many households continue to experience severe food inadequacy, standing at 4.8% (in 2017) [1], while 34.3% (in 2015) [7] live under the food poverty line.

Tuberculosis (TB), with 380 new cases per 1000 in 2016, is the leading cause of death in South Africa [11], and can be marginalizing, with numerous social and health consequences. Individuals with TB are often stigmatized [12] by peers (e.g., rejected by partners), within households (e.g., expulsion from homes) and at work (e.g., loss of employment). TB-stigma hampers health-seeking behavior [13], treatment initiation [14], and adherence to care [15], with potential consequences for the emergence of highly resistant strains to the standard antimicrobial isoniazid/rifampicin treatments. Incidence rates of TB and multidrug-resistant tuberculosis (MDR-TB) are 567 (includes HIV + TB) and 25 per 100,000 population, respectively [16], with the disease accounting for the largest percentage of total mortality in 2016 (6.5%) in South Africa [17]. According to the Global Burden of Disease Study 2015, TB is the fifth leading cause of years of life lost [18] and disability-adjusted life-years in South Africa [19]. With an estimated 30.9% case fatality rate [20], MDR-TB is, in some cases, virtually untreatable and deadly, which further exacerbates the downward cycle of TB-stigma and poor treatment adherence. In KZN, the prevalence of MDR-TB was 2.9% during 2012–2014 [21], and continues to be a major public health challenge.

Given the serious nature of the disease, exposure to MDR-TB can be a psychologically devastating and life-altering experience in the absence of adequate support mechanisms [22], posing a long-term threat to essential human security, particularly food security. In South Africa, the lack of mental health support is concerning, with only 25% of individuals with mental disorders having received treatment within a 12-month period [23]. KZN is the epicenter of the country’s MDR-TB epidemic, and has the largest share of poverty in South Africa (24.4%) in 2015 [7], as well as a high mental health treatment gap of approximately 80%, according to one estimate [24]. Although there are few quantitative estimates for South Africa, the treatment gap for major depression can range from 79 to 93% for low- and middle-income countries [25]. The estimated prevalence of (current) MDE in South Africa is 4.9% [26], while workplace-related economic loss due to depression is also estimated to be 4.9% of gross domestic product [27]. The treatment gap for depression co-morbid with HIV has been cited as a serious challenge [28].

According to several systematic reviews, the link between mental illness and food insecurity is well-documented globally [5, 6], but a significant gap in research remains that is relevant to the challenges endured by individuals with MDR-TB in Saharan Africa. In this current study, we investigated the role of MDE in household food insecurity among individuals with MDR-TB in KZN. This investigation was driven by a conceptual framework that posits that the link between food insecurity and chronic disease is a cyclic/bi-directional phenomenon [29]. Based on this framework and the notion of social drift which posits that clinical features in mental illness contribute to a downward socio-economic trajectory [30], and guided by previous works [31–33] that investigated the impact of depression as a predictor, we hypothesized that major depression among individuals with MDR-TB poses a “health shock” that contributes to household food insecurity.

## Methods

### Study design and participants

We prospectively enrolled and interviewed those with microbiologically confirmed MDR-TB who were consecutively admitted to a referral-based specialized TB hospital in KZN between September 2015 and October 2016. The inclusion criteria were consenting adult individuals (defined as aged 21–59 years) with no prior history of MDR-TB treatment. This study utilized data from an investigation that monitored neurocognitive impairment in participants with MDR-TB, requiring basic literacy to participate in the assessment and excluding individuals (*n* = 90) without primary school level education. Ethical approval was obtained from the University of KwaZulu-Natal’s Biomedical Research Ethics Committee. Five potential study participants refused to be involved in the investigation, and written informed consent was obtained from all others. Trained study personnel obtained socio-demographic data and administered the standardized questionnaire (including Household Food Insecurity Access Scale) through a face-to-face structured interview. Information on participants’ HIV status was obtained from the National Health Laboratory Service based at the study site.

### Household food insecurity

The outcome of the study was household food insecurity, which was measured using the Household Food Insecurity Access Scale version 3 [34]. As a widely used scale in developing countries, including studies in KZN with [35] and without HIV [36–38], HFIAS consists of a culturally invariant set of nine questions covering three domains related to household food insecurity: (1) anxiety/uncertainty about food supply, (2) altering quality of the diet, and (3) insufficient food intake. The response to each question was based on four choices (no = 0, rarely = 1, sometime = 2, often = 3). The composite scores were classified into food secure, insecure without hunger, with moderate hunger, and with severe hunger, according to the scoring guideline [34]. As the instrument pertains to food insecurity in the preceding 30 days, HFIAS was only administered to newly admitted study participants with less than a month of inpatient stay.

### Major depressive episode

Major depressive episode (MDE) was the main covariate of our study and was diagnosed using the Mini International Neuropsychiatric Interview version 6.0 (MINI 6.0) [39], a structured diagnostic schedule utilizing DSM-IV criteria. Research assistants underwent training by a South African psychiatrist for the MINI. In addition to HFIAS and MINI, socio-demographic information (e.g., gender, age, education, marital status, income categories and social grant status) was obtained through face-to-face interviews. We also collected (and included for analysis) data on preference in community residence, given its relevance to MDR-TB treatment [40].

### Data analysis

The analysis consisted of two components: first, descriptive statistics summarized the participants’ socio-demographic and clinical status. Second, logistic regression models were fitted to assess the association between MDE and household food insecurity while adjusting for socio-demographic and clinical status. We generated a final outcome measure that combines mild to severe insecurity categories to classify household food insecurity to fit the logistic regression models. STATA 15 was used for the analyses, with the model fit being assessed using the Akaike’s Information Criterion [41], where the lower values indicate a better fit.

## Results

### Socio-demographic and clinical status

The socio-demographic characteristics of the 141 MDR-TB study participants are presented in Table 1. Highlighting some results: most study participants were female (*n* = 110; 78.01%), black (*n* = 137; 97.16%), and had not completed secondary level education (*n* = 72; 51.06%). The prevalence of household food insecurity was 21.01% (*n* = 29), with approximately a third receiving a social grant (*n* = 41; 29.08%). The overwhelming majority were HIV+ (*n* = 124; 87.94%), and the proportion meeting MDE diagnostic criteria was 11.35% (*n* = 16).

**Table 1:** Socio-demographic status and clinical profiles of MDR-TB study participants (*N* = 141)

	Overall	
	*n*	%
Gender		
Female	110	78.01
Male	31	21.99
Age category		
21–29	47	33.33
30–39	55	39.01
40 +	39	27.66
Education		
< Grade 12	72	51.06
≥ Grade 12	69	48.94
Marital status		
Married/stable partner	81	57.45
Casual partner	14	9.93
No relationship/partner	46	32.62
Income categories		
Less than R1000 per month^a^	89	63.12
Greater or equal to R1000 per month	52	36.88
Social grant income		
No	100	70.92
Yes	41	29.08
Preference for continued living in one’s current community		
Strong/moderate preference to stay	107	75.89
No preference	25	17.73
Strong/moderate preference to leave	9	6.38
HIV status		
Positive	124	87.94
Negative	17	12.06
Major depression		
No	125	88.65
Yes	16	11.35
Food insecurity^b^		
Food secure	109	78.99
Insecure without hunger	3	2.17
Moderate hunger	6	4.35
Severe hunger	20	14.49

^a^The upper poverty line for South Africa was R992 in 2015 [42]

^b^Three missing response in HFIAS

### Association between MDE and household food insecurity

The results of the regression analysis are indicated in Table 2. The odds of household food insecurity, according to our final model (labeled model 2), were significantly higher [adjusted odds ratio (aOR) = 4.63, 95% CI 1.17–18.38] in individuals with MDE (compared to those without MDE). Examination of other covariates from the same model indicated that the odds of household food insecurity were significantly higher among those who were female (aOR 6.29, 95% CI 1.13–35.03), in the youngest age group (aOR 8.86, 95% CI 1.69–46.34), had low educational attainment (aOR 6.19, 95% CI 1.70–22.59) and were in receipt of a social grant (aOR 7.60, 95% CI 2.36–24.48). The strong moderate preference for continuing to live in one’s current community was associated with lower odds of insecurity, but was not statistically significant (*p* = 0.06). The Akaike’s Information Criterion (AIC) of the full model (model 2) was 124.23. Although model fit value of model 1 was lower, the significance of MDE under both models remain the same. The model 2 was retained and its findings reported due to the potential importance of income in our study.

**Table 2:** Adjusted logistic regression analysis results on the association between major depression and household food insecurity

	Model 1		Model 2 (final)	
	aOR	SE	aOR	SE
Major depression				
[No]				
Yes	4.16**	2.86	4.63**	3.26
Gender				
[Male]				
Female	5.59**	4.77	6.29**	5.51
Age category				
[40 +]				
21–29	7.67***	6.43	8.86***	7.48
30–39	2.28	1.8	2.08	1.63
Education				
[≥ Grade 12]				
< Grade 12	5.07***	3.06	6.19***	1.09
Marital status				
[Married/stable partner]				
Casual partner	1.31	1.17	1.51	1.37
No relationship/partner	0.95	0.57	0.98	0.59
Preference for continued living in one’s current community				
[No preference]				
Strong/moderate preference to stay	0.31	0.20	0.30	0.19
Strong/moderate preference to leave	0.93	1.06	0.88	0.97
Social grant income				
[No]				
Yes	6.83***	3.98	7.60***	4.54
Income categories				
[Greater or equal to R1000 per month]				
Less than R1000 per month			1.85	1.27
Model fit				
AIC	123.04		124.23	

**p < 0.05, ***p < 0.01. Above analysis based on *n* = 138 due to HFIAS. Reference group in bracket

## Discussion

The purpose of this study was to examine the association between major depressive episode and household food insecurity among individuals with MDR-TB from an area considered to be at the forefront of the outbreak in sub-Saharan Africa. Two important points emerged from our investigation. First, we found the proportion of severe food insecurity (14.49%) in individuals with MDR-TB to be markedly higher than that in the general South African population (4.8%) [1]. Second, the results of our study suggest an independent association between MDE and household food insecurity in this sample population, even after controlling for important measures of socio-economic status. Our study has also shown considerable gendered (i.e., female) and socio-economic dimensions (i.e., young, poorly educated, and in receipt of social grant) relating to food insecurity in South Africa.

Our main finding is consistent with various investigations [32, 33, 43–46], which found that depression/depressive symptomatology had a significant effect on household food insecurity. Our investigation on food insecurity was based on the notion of ‘health shocks’ [47], which is mainly found in the health/labor economics literature. Health shocks place the dual financial burden on individuals to bear the costs of medical treatment and income loss [48] due to under/unemployment. The length of inpatient stay for MDR-TB in South Africa may last 3–6 months (if not longer, until confirmed to be non-infectious [49]), while a major depressive episode (MDE) may become a chronic illness that requires life-long pharmacotherapeutic intervention and psychotherapy [50]. MDR-TB and/or MDE, therefore, places undue economic hardship on many affected persons, with their negative economic effects being particularly pronounced among the socio-economically vulnerable who have limited coping resources (e.g., social protection).

Notably, many studies about the role of depression and food insecurity have been conducted among vulnerable and marginalized groups, such as pregnant women [32, 33, 43], young people from informal settlements [51], the economically challenged elderly [44], low-income first-time mothers [45], and HIV+ individuals with substance use challenges [46]. In our results, we found that being socio-economically disadvantaged (e.g., female, young adult, low education, and social grant recipient) was a significant correlate of food insecurity. In the light of these findings, we thus argue, as one plausible explanation, that the added burden of MDE exacerbates the vulnerability of MDR-TB in a social drift towards eventual food insecurity.

The 12-month prevalence of MDE in our MDR-TB sample (11.35%) was higher than the South African general population estimate of 4.9% [26]. However, our estimate was lower than the prevalence of depressive episodes among individuals with TB reported from the World Health Survey (23.7%) (derived from community-based data from 48 low- and middle-income countries) [52]. It is possible that the lower than expected rate of depression in our study may relate to the fact that our participants were a *clinical* rather than *community* sample. Our study participants were receiving intensive inpatient treatment for MDR-TB at a specialized TB hospital, which may have had a positive impact on their depressive symptomatology.

The limitations of the investigation are the cross-sectional and modest sample size associated with our study design, which precludes any causal inference about the effect of depression on food insecurity. Our study is theory-driven (i.e., “health shock” among the socially marginalized), but we also acknowledge the possibility of reverse causation (effect of food security on depression), a point also considered in a recent study from KZN that found a significant association between food insecurity and depression among refugee and migrants [53]. As noted by others [54], and based on our literature review, a growing body of evidence implicitly assumes that food insecurity impacts on health, rather than the reverse. A stronger case for causal inference/temporal order, whether the main study predictor was depression or household food insecurity, requires a longitudinal investigation. Ideally, establishing a bidirectional link between food insecurity and depression would require an analytical model, such as structural equation modeling, which was not possible in this study due to the limited sample size [55]. Further, we acknowledge that the inpatients who were extremely ill due to MDR-TB (which may also be linked to depression, cognitive impairment and substance use) may not have participated in our investigation, thereby limiting generalization of our results. Finally, we did not have data on employment status of our inpatient sample which is a limitation as receipt of a social grant is not a proxy for unemployed status.

Notwithstanding the limitations, the significant association between depression and food insecurity among people with MDR-TB highlights the multifaceted health and social challenges that affected individuals who undergo in a setting considered to be at the epicenter of the MDR-TB epidemic in sub-Saharan Africa. Our current study comes at a time when such complex development challenges are further complicated by the growing threat to food security due to climate change in South Africa [56]. This will require sustainable livelihood approaches in research that not only focuses on food production, but also emphasizes factors that influence people’s food accessibility and utilization, such as poverty and health [57]. To our knowledge, there are few studies of MDR-TB in sub-Saharan Africa with a strong mental health component that includes a diagnosis of mental disorder. Our study underscores the need to end the cycle of food insecurity and chronic disease (MDE and MDR-TB) by providing sufficient social protection to buffer against health shocks, as well as accessible high-quality mental health care services for the socially disadvantaged populations of South Africa.
